# Diphtherial Relapses

**Published:** 1907-10-26

**Authors:** 


### POINTS IN TREATMENT.

### DIPHTHERIAL RELAPSES.

Diphtheria, though in many respects a specific fever, differs from most others in that one attack does not protect the patient from a subsequent one. The immunity conferred by one attack is comparatively short-lived, so that second attacks are not very uncommon. It is not, however, of second attacks at a considerable interval after the first that it is proposed to speak here, but of true relapses: that is to say, fresh outbreaks of diphtheria in patients who are not yet fully convalescent from a previous one. Such relapses are not very common, but there are certain points about them which make them important. Various authorities have recorded statistics upon the subject, and nearly all agree in putting the frequency of true relapses, as distinct from second attacks, at something between 1 and 2 per cent. of all cases of diphtheria.

It is not, of course, every sore throat that arises during convalescence that can at once be put down as a relapse; follicular tonsillitis, recurrent sore throat, and so forth, must be excluded. The persistence of diphtheroid bacilli for weeks after the acute illness is past does not constitute a relapse either; in a real relapse the patient develops an illness very similar to the first attack, with fresh exudation of typical membrance, usually affecting the same site as before, but occasionally attacking a different site. After faucial diphtheria, the relapse is usually faucial, and so on; though now and then a faucial attack may have a relapse in which the membrane is in the nose, and so forth.

In former days the intensity of the relapse used to be very much greater than that of the original illness, but since the introduction of serum treatment, the relapses have almost all been milder. They do not as a rule occur before the third week, and they may be as late as the twelfth or even fourteenth.

Treatment.

The most important point about them is in regard to treatment. Relapse cases react with extreme sensitiveness to antidiphtheritic serum, so that whereas 20,000 units of the latter are well within the limit of dosage for the first attack, the dose for a relapse in a case where serum has been used for the original attack should not exceed 8,000 units. Even when this comparatively small dose is employed the serum is very liable indeed to cause erythematous or urticarial eruptions on the skin, and joint pains or "weary bones," with some pyrexia. J. D. Rolleston lays stress upon this, and points out that whereas the serum rash seldom occurs before the eighth or ninth day after an original injection, the trouble comes on within a few hours of a re-injection in many cases, if the interval between injection and re-injection is more than ten days. Moreover, there may be extensive œdema as well as the rash, and occasionally rigors, vomiting and collapse have been observed.

This being so, and seeing that the relapse is usually mild when serum has been given for the original attack, it would seem that relapse cases are best treated without serum; in any case, it is very important to give a much smaller dose of serum for a relapse than that which is required for the original diphtheria.

